# Global Trends in the Relationship Between Chronic Air Pollution Exposure, Physical Activity and Lung Function in Youth Aged 5–18 Years With and Without Asthma: A Systematic Review

**DOI:** 10.1186/s40798-025-00856-3

**Published:** 2025-05-21

**Authors:** Samanta Gudziunaite, Kelly A. Mackintosh, Gwyneth A. Davies, Kathryn A. Jordan, Paul D. Lewis, Chris J. Griffiths, T. Alexander Swain, Melitta A. McNarry

**Affiliations:** 1 Applied Sports, Technology, Exercise and Medicine (A-STEM) Research Centre, Swansea University, Swansea, UK; 2 Swansea University Medical School, Swansea University, Swansea, UK; 3 Swansea University School of Management, Swansea, UK; 4 Wolfson Institute, Queen Mary University of London, London, UK

**Keywords:** Air quality, Health outcomes, Paediatrics, Children, Adolescents, Environmental exposures

## Abstract

**Background:**

Children are more susceptible to air pollution due, at least in part, to their less-developed respiratory systems and higher respiratory rates. Although the health benefits associated with physical activity are indisputable, there is considerable debate regarding whether increased exposure to, and deeper inhalation of, air pollution while being physically active negates such health benefits.

**Objectives:**

The aim was to explore the relationship between air pollution and lung function and the influence of asthma status and physical activity in children and adolescents.

**Methods:**

Six databases were searched following PRISMA guidelines with no date restrictions: PubMed, Web of Science, MEDLINE, EMBASE, SPORTDiscus, and Cochrane Central Register of Controlled Trials (CENTRAL). Studies were included if they: i) studied children and adolescents (5–18 years); ii) were peer-reviewed; iii) were available in the English language; and iv) reported data using previously validated tools.

**Results:**

From 12,161 original records, 16 studies were included in this review. The most widely examined pollutants were particulate matter PM_2.5_–PM_10_, ozone (O_3_), nitrogen dioxide (NO_2_), nitrogen oxide (NO_X_), carbon monoxide (CO), and sulphur dioxide (SO_2_). Increased exposure to various air pollutants, particularly during outdoor physical activity, resulted in lung function deficits. This was especially evident in children and adolescents with asthma, dependent on the specific air pollutant. There was a consensus that forced expiratory volume in one second (FEV_1_) and forced vital capacity (FVC) decreased as air pollution concentrations increased. Notably, there was a reduction in FEV_1_ at both three- and four-days post-exposure to CO, PM_10_, and NO_2_.

**Conclusions:**

There is a pressing need to reduce the impact of air pollution on lung function to improve health and realise the full benefits of physical activity. Given the potent and potentially long-term effects of air pollution, governments and local authorities must continue to reduce air pollution concentrations to improve the current and future health of populations globally.

**Key Points:**

- Increased exposure to air pollutants results in impairments of children’s and adolescents’ lung function, with the most pronounced effects observed three-to-four days post-exposure. This delayed impact suggests a prolonged risk of respiratory impairment following exposure, but further work is required to fully elucidate the timeline and associated dose-response relationship.
- The limited evidence available suggests that physical activity levels may be lower during periods with high air pollution concentrations, particularly in those living in urban areas or near roads. This is especially concerning for children with asthma, who are at a greater risk of experiencing poorer lung function due to the combined effects of reduced physical activity and increased pollutant concentrations.
- Physical activity during periods of high air pollution concentrations is tentatively suggested to deleteriously influence lung function in children and adolescents.

**Supplementary Information:**

The online version contains supplementary material available at 10.1186/s40798-025-00856-3.

## Background

Characterised by a persistent cough, wheezing, chest tightness, and breathlessness, asthma is a multifactorial, noncommunicable disease (NCD) and a public health concern, affecting approximately 339 million people globally [1, 2]. Regular physical activity is associated with numerous health benefits, such as improved physical, social, and mental health, and overall well-being [3–5], and it is typically recommended to manage and minimise asthma-related complications. However, moderate-to-vigorous physical activity (MVPA) may engender greater exposure to, and intake of, air pollution. Indeed, reducing exposure to indoor and outdoor air pollution is becoming a priority as children and adolescents who spend more time outdoors during times with higher air pollution concentrations will likely have lower age-related increases in peak flow measurements and deficits in lung function [6–10].

The World Health Organization (WHO) recently highlighted that 93% of children worldwide breathe polluted air that exceeds WHO guidelines for particulate matter with an aero diameter of 2.5 µg/m^−3^ or less (PM_2.5_), particulate matter with an aero diameter of 10 µg/m^−3^ or less (PM_10_), ozone (O_3_), nitrogen dioxide (NO_2_), sulphur dioxide (SO_2_), and carbon monoxide (CO) [11], with these pollutants suggested to be the most harmful in terms of lung function [12–15]. Children are also potentially at risk of greater impacts of air pollution due to their immature bronchi and associated epithelial cells [16, 17]. Furthermore, compared to adults, children are at risk of a greater exposure to and intake of air pollutants due to their higher respiratory rate [18] and shorter stature which decreases the distance from vehicle exhausts where pollutants tend to reach peak concentrations [19–21].

The deleterious consequences of air pollution inhalation may be related, amongst other things, to the promotion of airway sensitivity, mucosal damage, and allergen penetration [22, 23]. Specifically, gaseous pollutants may induce hyperosmolarity and, therefore, airway hyperresponsiveness [24, 25], whilst exposure to particulate matter was found to cause microtrauma to the airway epithelium, leading to localised inflammation [26, 27]. The specific impacts of particulate matter are dependent, in part, on the specific size—PM_10_ typically deposits in the lungs whereas PM_2.5_ penetrates the alveoli and ultrafine particulates enter the bloodstream and cause localised respiratory complications [28–30].

Air pollutants are a common irritant for those with asthma and have been associated with higher mortality [31–33], increased sensitisation to allergens [34], and impaired lung function [35]. Indeed, there are more frequent reports of wheezing/whistling, asthma exacerbations, more challenging asthma management, especially in those with poorly controlled symptoms, and increased hospitalisations when air pollution concentrations are high [36–41]. Furthermore, PM_2.5_ has been associated with the development of asthma and persistent wheezing in children, whilst reducing exposure to PM_2.5_ decreases asthma-related symptoms [42]. Indeed, McConnell et al. [43, 44] reported a higher incidence of new asthma diagnoses in physically active children in areas with high O_3_ concentrations, raising an important question as to whether the increased exposure engendered by being active in areas of high pollution potentially negates the benefits associated with the activity. Despite the implications of such suggestions, there remains little consensus as to the potential mediating effect of physical activity on the relationship between air pollution [45] and lung function. Consequently, the aim of this systematic review was to synthesise current evidence regarding the relationship between air pollution (CO, O_3_, PM_1_, PM_2.5_, PM_10_, NO_2,_ and NO_X_), physical activity (self-report or device-based), and lung function—measured by forced expiratory volume in one second (FEV_1_), forced vital capacity (FVC), and peak expiratory flow (PEF)—in children and adolescents with and without asthma.

## Methods

### Searches

The current systematic review was conducted according to the Preferred Reporting for Systematic Reviews and Meta-Analyses (PRISMA) checklist [46], with the review protocol registered on the PROSPERO International Prospective Register of Systematic Reviews (CRD42022307206).

The literature was originally searched until 15th March 2022 using the following sources, with no date restrictions for records: PubMed, Web of Science, MEDLINE, EMBASE, SPORTDiscus, and Cochrane Central Register of Controlled Trials (CENTRAL). The searches were subsequently periodically updated, with the most recent search conducted on 29th May 2024.

### Inclusion and Exclusion Criteria

To be included in this systematic review, studies had to: i) use validated tools for measuring lung function or physical activity (including country guidelines for acceptable quality spirometry and clearly defined accelerometer wear time or physical activity diaries/questionnaire); ii) be peer-reviewed; iii) be available in the English language; and iv) involve children and adolescents aged 5–18 years. The key focus of this review was outdoor air pollutants, specifically CO, NO_2_, NO_x_, O_3_, PM_1_, PM_2.5_, PM_10_, and SO_2._ All study designs were eligible for inclusion unless they were laboratory-based or involving animals. Gray literature and unavailable full texts were excluded. Search terms are described in Supplementary material 1.

This systematic review focused on lung function parameters, including FEV_1_, FVC, and PEF. Tools and methods for measuring physical activity included accelerometers and physical activity diaries/questionnaire.

### Effect Modifiers and Reasons for Heterogeneity

Due to the heterogeneity of the 16 studies included in this systematic review, it was not possible to conduct a meta-analysis and, therefore, potential effect modifiers could not be explored. It should be noted that the heterogeneity was indicative that there is currently little replication of findings and caution is therefore required in the interpretation of their results. This variation among the included studies stemmed from differences in study design, exposure measurements, and outcome definitions.

### Data Extraction

All papers were reviewed by two authors (SG and MAM) using a double-blind method, and any discrepancies resolved through a consensus meeting. Where a consensus could not be reached (*n* = 3), PL was consulted. One hundred and nine papers were retained for full-text screening, of which 14 could not be retrieved despite contacting the authors and 79 were excluded due to not meeting the inclusion criteria (*n* = 73) or being outside the scope of the review (*n* = 6).

### Study Quality Assessment

Data were extracted by SG and KAJ, with MAM verifying the relevant variable fields. The risk of bias was assessed using the standardised data extraction form [47] for the Mixed Methods Appraisal Tool [48]. Each study was subsequently assigned an overall quality score ranging from 1*, where 20% of the quality criteria had been met, to 5*, where 100% of the quality criteria had been met [47]. Questions considered whether participants were representative of the target population, if the measurements were appropriate to both the outcome and intervention (or exposure), if complete outcome data were reported, whether confounders were accounted for in the design and analysis, and during the study period and whether the intervention/exposure occurred as intended.

The certainty of the evidence was subsequently assessed according to the Synthesis Without Meta-analysis (SWiM) guidelines which were developed to guide reporting in reviews in which alternative synthesis methods to meta-analysis of effect estimates are used [49]. Table 1 highlights the grouping of the studies according to the SWiM grouping. The studies were grouped into two categories based on whether they took a dose–response or categorical approach to describe the relationship between air pollution and key outcome variables. Supplementary material 2 further describes the SWiM reporting items.

**Table 1:** Characteristics of the included studies

Study	Study design (year)	Sample size (boys/girls)	Age (years)	Asthma (yes/no)	PA (yes/no)	Country	Location (urban/rural)	Outcome †	Type of pollutant measured ††	Synthesis of results	Assigned SWiM group [49]	Quality assessment (MMAT) [47] †††
Aguilera et al. [59]	Cohort (2023)	12 (7/5)	6–12	Yes (*n* = *12*)	Yes (accelerometery)	USA	Urban	Physical activity behaviours	PM_2.5_, PM_10_, NO_2_, O_3_	During times of high air pollution concentrations children’s behaviour changed; time spent in MVPA decreased and sedentary behaviour increased. Specifically, PM_2.5_, PM_10_, and NO_2_ were positively associated with increased sedentary behaviour among children with asthma	2	*** (60%)
Avol et al. [58]	Cohort (2001)	110 (59/51)	10	No	No	USA	Both	FEV_1_, FVC, MMEF, PEF	O_3_, NO_2_ and PM_10_	Participants who moved to areas of lower PM_10_ showed increased growth in lung function compared to those who moved to areas of high PM_10_	2	**** (80%)
Dimakopoulou et al. [51]	Cohort (2020)	186 (93/93)	10–11	Yes (*n* = *21*)	No	Greece	Urban	FEV_1_ and FVC	O_3_ and PM_10_	A 10 µg/m^−3^ increase in O_3_ exposure was associated with lower FVC and FEV_1_ and small decreases in lung function	2	***** (100%)
Gao et al. [63]	Cross-sectional (2012)	2,060 (1,64/996)	8–10	Yes (*n* = *68*)	No	China	Urban	FEV_1_, and FVC	SO_2_, NO_2_, O_3_ and PM_10_	Long-term exposure to higher ambient air pollution concentrations (above 109 µg/m^−3^ for O_3_) was associated with lower lung function in schoolchildren, especially among boys	1	***** (100%)
Gilliland et al. [57]	Cohort (2017)	2,120 (1,105/1,015)	10–18	No	No	USA	Both	FEV_1,_ FVC and bronchitic symptoms	NO_2_, O_3_ and PM_10_	Respiratory symptoms at ten years and 15 years were significantly reduced by decreased exposure to NO_2_, O_3_ and PM _2.5–10_, with the greatest improvements associated with decreases in PM_10_. Greater improvements were reported in those with asthma than those without, for comparable changes in air pollution concentrations	2	***** (100%)
Hoek et al. [66]	Cross-sectional (1993)	83 (40/43)	7–12	No	Yes (training sessions)	Netherlands	Urban	PEF	O_3_ and NO_2_	High previous-day O_3_ exposure was associated with a lower PEF following 1-h physical exercise the next day	1	***** (100%)
Hwang et al. [60]	Cohort (2015)	2,941 (1,532/1,409)	12	Yes (*n* = *232)*	No	Taiwan	Both	FEV_1_ and FVC	PM_2.5_, O_3_, SO_2_ and NO_2_	Lower age-related increases in FVC and FEV_1_ were associated with increased exposure to PM_2.5_ and O_3_	2	***** (100%)
Karakatsani et al. [52]	Cohort (2017)	188 (93/95)	10–11	No	No	Greece	Urban	FEV_1,_ FVC, PEF and FeNO	O_3_, PM_10_ and NO_2_	A 10 µg/m^−3^ increase in weekly O_3_ was associated with decreased FVC and FEV_1_	2	***** (100%)
Li et al. [62]	Cohort (2020)	684 (374/313)	7–12	No	No	China	Urban	FEV_1_, FVC, PEF, and FEV_1_/FVC	PM_2.5_	A 1 µg/m^−3^ increment in average daily dose (ADD) of PM_2.5_ was associated with a decrease in lung function (FVC and FEV_1_), with a greater magnitude of effect in girls than boys	2	**** (80%)
Ntarladima et al. [53]	Cohort (2021)	638 (299/339)	8	No	No	Netherlands	Both	FEV_1_ and FVC	NO_2_, NO_x_, PM_2.5_ and PM_10_	Time-activity patterns (calculations based on a secondary dataset containing questionnaire data reporting on PA) and exposure to NO_2,_ PM_2.5_ and PM_10_ were correlated with decreases in lung function (FVC and FEV_1_)	2	**** (80%)
Roy et al. [61]	Cohort (2023)	250 (115/135)	9–12	No	No	Bangladesh	Urban	PEF	PM_1_, PM_2.5_, PM_10_, NO_2_, CO_2_	Children attending schools in dense neighbourhoods, or near roadside were exposed to higher concentrations of traffic related air pollutants and as such appeared to experience lower PEF values compared to those attending schools exposed to less PM_1_ and PM_2.5._ Most outdoor air pollution monitoring sites reported concentrations of pollutants above WHO recommended guidelines. However, the association between PEF and PM_1.0_, PM_2.5_ was very weak	2	*** (60%)
Spektor et al. [56]	Cohort (1990)	46 (33/13)	8–14	No	No	USA	Rural	FEV_1,_ FVC, PEF	O_3_	All children attended a summer camp, and via questionnaire, they recorded their daily activities and duration. Ambient O_3_ exposures elicit greater lung function deficits in active young people	2	**** (80%)
Suhaimi et al. [64]	Cross-sectional (2023)	115 (73/82)	7–11	No	Yes (daily activity diaries)	Malaysia	Urban	FEV_1_ and FVC	PM_2.5_, PM_10_, NO_2_, SO_2_, O_3_, and CO	Children who were exposed to high traffic related air pollution concentrations (particularly PM_2.5_) experienced deficits in FVC and FEV_1_ when compared to those living/studying in neighbourhoods with lower levels of air pollution. Those exposed to high air pollution concentrations also reported more frequent respiratory symptoms such as cough, phlegm, wheezing, and chest tightness	1	***** (100%)
Timonen et al. [54]	Cohort (2002)	33 (18/15)	12	Yes (*n* = *7*)	Yes (exercise challenge test)	Finland	Urban	FEV_1_ and FVC	PM_10_, NO_2_, CO and SO_2_	Increased black smoke PM_10_, NO_2_, and CO concentrations were associated with impaired baseline lung function	2	**** (80%)
Wang et al. [65]	Cross-sectional (2017)	71,768 (35,845/35,923)	7–18	No	No	China	Urban	FVC	PM_10_, NO_2_ and SO_2_	Children living in cities with higher annual average concentrations of PM_10_ experienced poorer lung function (FVC); A 10 µg/m^−3^ increase in PM_10_ was associated with a 0.0013 l decrease in FVC	1	***** (100%)
Zebrowska and Mankowski [55]	Cohort (2010)	103 (55/48)	14–16	No	No	Poland	Urban	FEV_1_, FVC, and MVV	PM_10_, NO_2_, CO and SO_2_	Exposure to air pollution was associated with reduced respiratory function	2	**** (80%)

^†^ CO, carbon monoxide; CO_2_, carbon dioxide; NO_2_, nitrogen dioxide; NO_x_, nitrogen oxide; O_3_, ozone; PM_2.5_, particulate matter with microns of 2.5 in diameter; PM_10_, particulate matter with microns of 10 in diameter; SO_2_, sulphur dioxide

^††^ FeNO, fractional exhaled nitric oxide; FEV_1_, forced expiratory volume in one second; FVC, forced vital capacity; MMEF, maximal mid-expiratory flow; MVV, maximal voluntary ventilation; PEF, peak expiratory flow

^†††^ Following Mixed Methods Appraisal Tool (MMAT) criteria by Hong et al. [47]. 1*, where 20% of the quality criteria had been met, to 5*, where 100% of the quality criteria had been met

## Results

### Study Characteristics

**Fig. 1:**
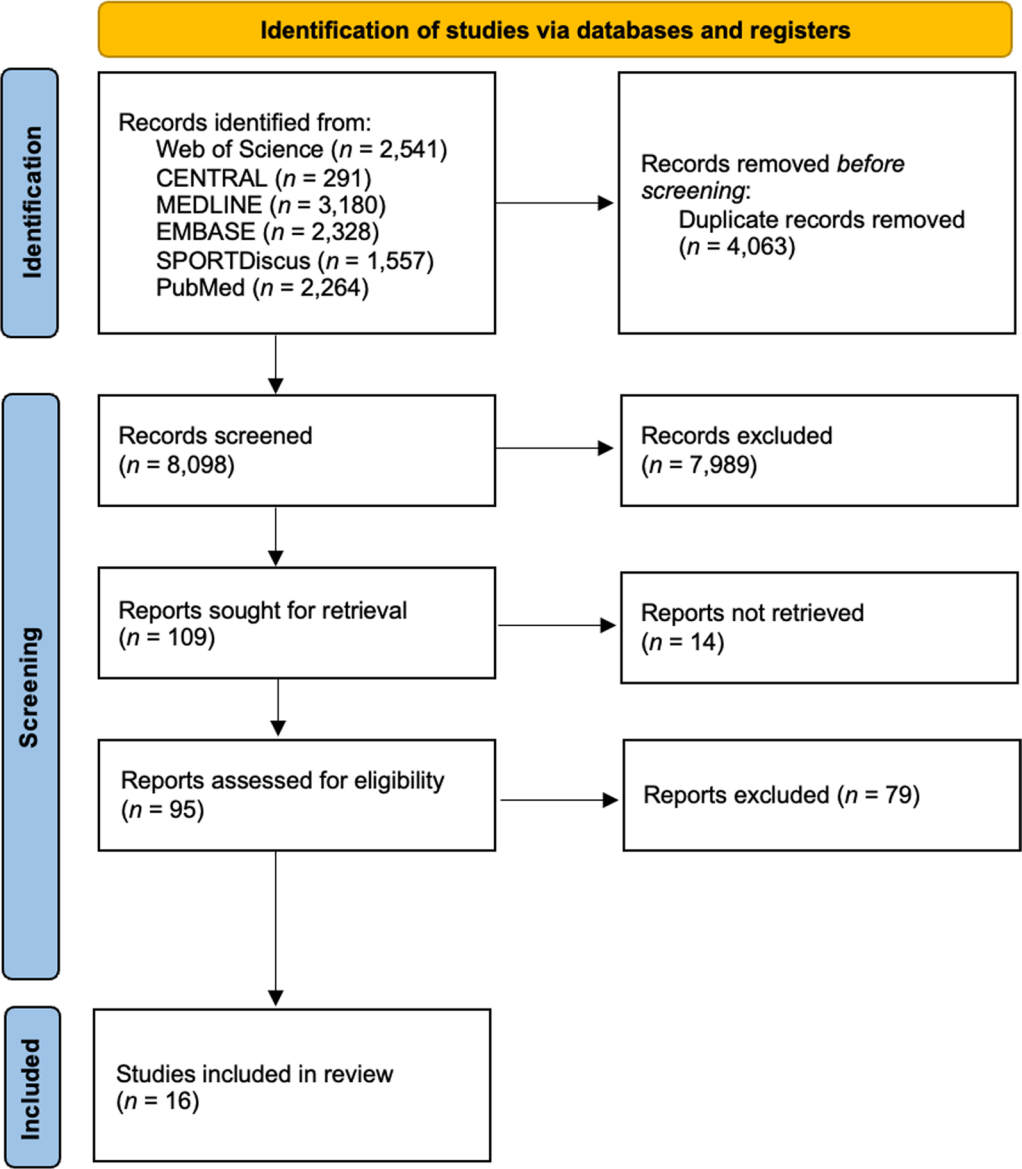
Study review process following the PRISMA guidelines

Following the initial search, 12,161 references were retrieved and transferred from EndNote library to the Rayann Web app [50]. Subsequently, 4,063 duplicates were removed, and 8,082 papers were excluded for not meeting the inclusion criteria (Fig. 1).

The studies included in the review are shown in Table 1 and consisted of four cross-sectional and 12 cohort studies from different cohorts. Five cohort studies were conducted in Europe [51–55], four in North America [56–59], and three in Asia [60–62]. Three cross-sectional studies were conducted in Asia [63–65] and one was conducted in Europe [66]. Eleven studies were conducted in urban areas [51, 52, 54, 55, 59, 61–66], one in a rural area [56], and four in a combined urban and rural area [53, 57, 58, 60]. Overall, there were 81,337 participants, with most studies including ≤ 500 participants. Data collection for the included studies ranged from 1989 to 2023. Following the risk of bias assessment (MMAT), eight studies were rated 100% [51, 52, 57, 60, 63–66], six as 80% [53–56, 58, 62], and the remaining two as 60% [59, 61] due to incomplete outcome data being available.

### Synthesis of Findings

**Fig. 2:**
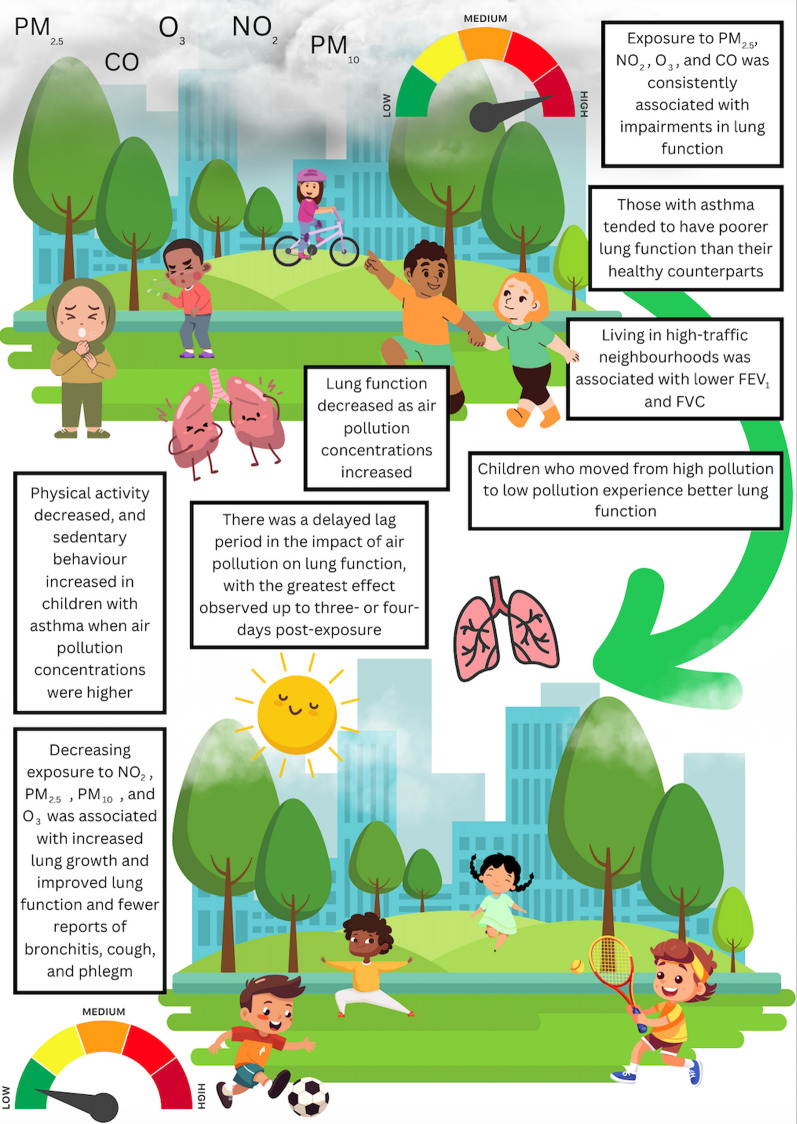
An infographic to demonstrate the findings of the systematic review

Living in less polluted areas or having lower long-term exposure to pollutants was associated with higher overall lung function [57], with relocating from areas of high to low air pollution associated with improvements in lung function [58] (Fig. 2). Specifically, Gilliland et al. [57] found that in children with asthma, reductions in NO_2_, PM_2.5_, and PM_10_ exposure were associated with improvements in FEV_1_ and FVC over four years. Furthermore, the decreased air pollution exposure was associated with fewer reports of respiratory symptoms, including cough and phlegm. Spektor et al. [56] reported that in active youth short-term exposure to air pollution (O_3_ in the previous hour) resulted in reductions in FVC and FEV_1_. Similarly, Timonen et al. [54] found that high concentrations of PM_2.5_, NO_2_, and CO were associated with impairments in lung function before cycling. There also appeared to be a delayed lag period in the impact of air pollution on lung function, with the greatest effect observed up to three days post-exposure, which was consistent for CO, PM_10_, and NO_2_.

### Long-Term Effects of Air Pollution and Air Quality Variability on Lung Function

Several studies have investigated the associations of air pollution and lung function. For example, Karakatsani et al. [62] concluded that a 10 µg/m^−3^ increase in O_3_ was associated with a decrease in FEV_1_ by 0.03 L (95% CI: − 0.05, − 0.01) and a decrease in FVC by 0.01 L (95% CI: − 0.03, − 0.003), as well as a 19% increase in at least one respiratory-related symptom. Hwang et al. [60] conducted a cohort study in Taiwan and reported an association between changes in air pollution and lung function with deficits in FVC and FEV_1_ associated with increased exposure to PM_2.5_ and O_3_ [60]. Additionally, boys were more susceptible to reduced lung function compared to girls following increased O_3_ exposure. O_3_ had a negative effect on FVC and FEV_1_, with spirometry values of − 0.05 L (95% CI: − 0.09, − 0.02) and − 0.06 L (95% CI: − 0.09, − 0.03) for boys and − 0.04 L (95% CI: − 0.07, − 0.02) and − 0.05 L (95% CI: − 0.07, − 0.02) for girls, respectively. A cross-sectional study by Gao et al. [63] that categorised participants into low-, medium- and high-pollution locations found no difference in asthma prevalence across districts, but higher adverse effects on lung flow measures where deficits ranged from 12.2% to 58.2%. The same study [63] reported that boys in the high-pollution district had a significantly lower average FEV_1_ compared to those in the low- and medium-pollution districts. Specifically, FEV_1_ in the high-pollution district was 3.0% lower than in the low-pollution district and 3.5% lower than in the medium-pollution district. No differences were observed for girls. Similarly, Wang et al. [65] concluded that decreasing exposure to NO_2_, PM_2.5_–PM_10_, and O_3_ was associated with improved lung function and fewer reports of bronchitis, cough, and phlegm among children in urban areas of China. The relationship between air pollution and lung function was consistent irrespective of season, despite the influence of seasonality on both factors [65]. In contrast, during an eight-month cohort study in Bangladesh, Roy et al. [61] observed a negative trend in PEF with increasing exposure to PM_1_ and PM_2.5_, although the relationships failed to reach statistical significance. Lastly, Suhaimi et al. [64] conducted a cross-sectional study in Malaysia where they found that children attending school in heavy traffic neighbourhoods were exposed to higher concentrations of PM_2.5_ and demonstrated lower FVC and FEV_1_ readings. Children living in high traffic areas (PM_2.5_ > 56.50 µg/m^−3^ and PM_10_ > 67.63 µg/m^−3^) had a fourfold greater probability of a lower FEV_1_ and FVC, and a sevenfold greater probability of a lower FEV_1_/FVC in comparison to their counterparts who resided in low traffic areas.

### Delayed Lag Period

Regarding lung function and the long-term implications, one study by Timonen et al. [54] found a delayed lag period in the impact of air pollution on lung function that varied according to the pollutant, with the greatest effect manifesting up to three days post-exposure, which was consistent for CO, PM_10_, and NO_2_. Spektor et al. [56] reported that in active youth previous day exposure to O_3_ resulted in reductions in FVC and FEV_1_ by -0.38 ± 0.11 ml and − 0.43 ± 0.08 ml. Hoek et al. [66] also found a negative trend for PEF after training sessions following previous day O_3_ exposure. It is also pertinent to note that while defined as ‘exercise’ the training sessions included athletics, baseball, korfball, tennis, or short games and therefore, they could be more in line with the definition of MVPA.

### Physical Activity and the Relationship between Air Pollution and Lung Function

Few studies have considered the role of physical activity in the relationship between air pollution and lung function, with the majority of studies focussing specifically on the influence of acute exposures to air pollution during exercise [54, 56, 57, 66]. Hoek et al. [66] examined the effect of outdoor training sessions and O_3_ on children’s lung function and reported that there was a lack of association between children’s PEF before and after sessions following exposure to O_3_. Only one study used accelerometery data to investigate the effect of air pollution exposure on the physical activity levels in children with asthma [59], reporting that PM_2.5_, PM_10_, and NO_2_ were negatively associated with time spent in MVPA. For example, for every interquartile range increase in 96-h mean concentration for PM_2.5_, PM_10_, and NO_2,_ the percentage of time children spent in MVPA decreased by − 3.45% (95% CI: − 5.00, − 1.90), − 1.59% (95% CI: − 2.37, − 0.18), and − 1.35% (95% CI: − 2.62, − 0.09), respectively. Conversely, time spent sedentary was positively correlated with air pollution concentrations, with increases of 3.43% (95% CI: 1.78, 5.09), 1.51% (95% CI: 0.69, 2.34), and 1.52% (95% CI: 0.25, 2.79) for PM_2.5_, PM_10_, and NO_2_, respectively. Spektor et al. [56] reported similar findings - among active youth, short-term exposure to air pollution (O_3_ in the previous hour) resulted in reductions in FVC and FEV_1_ of -1.53 ± 0.38 ml and -1.60 ± 0.30 ml, respectively.

The location in which children spent most of their time in routine daily activities, such as attending school, being at home, spending time in daycare, or tuition centres determined their exposure to, and intake of, air pollutants. In Suhaimi et al. [64], the utilisation of daily activity diaries facilitated the evaluation of how physical activity levels might influence exposure to air pollutants due to location and the duration of time spent in these locations. Children living in highly polluted neighbourhoods typically spent more time doing outdoor physical activities than their counterparts living in low pollution neighbourhoods who spent more time at home. The children who were more physically active outdoors in high-pollution neighbourhoods were four times more likely to experience abnormalities in their FEV_1_ or FVC readings (as defined within the study) when compared to their counterparts in low-pollution neighbourhoods. In contrast, Timonen et al. [54] reported small changes in lung function following an outdoor exercise test during periods of low pollution (approximately a 5% decrease in FEV_1_). However, when the exercise was repeated during an episode of high PM_10_ and SO_2_, there was no significant association between air pollution and changes in lung function.

### Air Pollution and the Role of Asthma Status

A cross-sectional study found that asthma prevalence in low-, medium-, and high-pollution districts was 2.6%, 2.1%, and 3.3% [63]. Initially, it appeared that children with asthma in the high-pollution district had poorer lung function than those in the low- and medium-pollution districts, irrespective of sex, and to a greater extent than their non-asthma counterparts. However, upon further analysis similar associations were observed among children with and without asthma, indicating no difference depending on asthma status. Suhaimi et al. [59] found that physical activity decreased and sedentary behaviour increased in children with asthma when air pollution concentrations were higher but lung function was not reported. While Suhaimi et al. [59] did not specifically focus on asthma status, they concluded that children living in high-traffic neighbourhoods were more likely to report respiratory symptoms, including cough (eightfold), phlegm (sevenfold), wheezing (fourfold), and chest tightness (fourfold) when compared to their counterparts living in low-pollution neighbourhoods. In contrast, Hwang et al. [60] found that while children with asthma had slightly worse lung function than their counterparts, the overall impact of air pollution was similar regardless of asthma status. Despite collecting data on asthma status, Dimakopoulou et al. [51] were not able to explore the influence of long-term O_3_ exposure in children with asthma due to the low number of participants (*n* = 15 with doctor-diagnosed asthma, total sample size *n* = 186). Another study demonstrated that declines in air pollution exposure correlated with reductions in respiratory symptoms for all individuals, but those with asthma benefited more than their healthy counterparts for a given change in air pollution. For example, Gilliland et al. [57] reported that respiratory symptoms at 10 and 15 years were significantly reduced when exposure to NO_2_, O_3_, PM_10_, and PM_2.5_ decreased, with the greatest improvements being associated with decreases in exposure to PM_10_.

## Discussion

All 16 studies included in this systematic review, which focused specifically on the effects of O_3_, PM _2.5–10_, and NO_2_, reported that increased exposure to these air pollutants was associated with lower lung function (FEV_1_, FVC, or PEF). The key findings were that: (i) those with asthma exposed to comparable concentrations of pollutants tended to have poorer lung function than their healthy counterparts; (ii) lung function decreased as concentrations of NO_2_, PM_2.5_, PM_10_, and O_3_ air pollutants increased; (iii) decreasing exposure to NO_2_, PM_2.5_, PM_10_, and O_3_ air pollutants resulted in fewer reports of respiratory symptoms; and (iv) among children with asthma, when air pollution concentrations were high, physical activity levels decreased, and sedentary behaviour increased (Fig. 2).

### Physical Activity, Lung Function, and Asthma

While physical activity is known to offer numerous health benefits, exposure to and/or intake of higher concentrations of pollutants may counteract the otherwise positive benefits of being physically active [67, 68]. Indeed, increased concentrations of air pollution are associated with lower lung function [16, 69], which may represent a physical barrier to physical activity [70], regardless of asthma status. For example, pedestrian volume and the choice of location for physical activity (outdoor green spaces vs indoor spaces) fluctuate based on air pollution levels [71]. While reduced MVPA during times of high air pollution may be associated with decreased lung function, other factors, such as discomfort or perceived risk, likely play a role in an individual’s decision on where they spend their time. Jiang et al. [72] observed that citizens underutilise parks and outdoor spaces when air pollution concentrations change from moderate to high. However, despite the health risks associated with air pollution exposure, a proportion (41–64%) of individuals surveyed [72] agreed that they would continue to visit these spaces, suggesting the relationship between physical activity and lung function is complex. This raises further questions regarding the public’s awareness of air quality dangers and this may be particularly true for children with asthma.

Children with asthma may be more inclined to engage in sedentary behaviours when air pollution concentrations are high, likely to avoid respiratory symptoms that might worsen with MVPA [59, 73, 74]. Although qualitative research has explored children’s perceptions surrounding air pollution, physical activity, and asthma [75], there remains a gap in understanding youths’ behaviours and approaches towards decision-making in such scenarios, particularly regarding their capacity and willingness to implement changes, as well as what they perceive as beneficial. Such changes require comprehensive strategies—and stakeholder engagement and patient and public involvement—to address both environmental and psychological factors when considering effective physical activity interventions aimed towards reducing children’s exposure to and intake of air pollution whilst maximising physical activity and implementing positive long-term behaviour change [76, 77].

Previous research found that harmful particulates can exacerbate asthma by further reducing lung function and irritating the airways [70, 78]. Tiotiu et al. [78] specifically highlighted that while air pollution negatively impacts all children, those with bronchial hyperresponsiveness or allergen sensitisation, particularly children with asthma, are at higher risk of impaired lung function. This is congruent with the findings of the current systematic review, where three studies investigating the effect of air pollution exposure on lung function in children with asthma consistently showed that children with asthma had worse lung function than their counterparts without asthma, likely due to heightened sensitivity to variations in pollutants [57, 60, 64]. Indeed, lung function was lower among children with asthma living in areas with more pollution than those living in less polluted areas [58, 63]. While the evidence suggests that children with asthma are often more vulnerable to air pollution exposure, most research has focused on children without asthma, leaving this high-risk group underrepresented. Understanding how children with asthma navigate decision-making about physical activity during times of high air pollution may provide further insights into the environmental and psychological factors influencing their behaviours and willingness to be physically active.

Furthermore, it was found that declines in lung function were a particular cause for concern when air pollution concentrations were elevated at roadside locations [64]. In one study that utilised questionnaires, participants collectively reported that lung function deteriorated in accordance with physical activity levels, suggesting that physical activity might mediate the relationship between air pollution and lung function [56]. Multiple studies have also associated higher concentrations of PM_2.5_ and other pollutants, such as SO_2_, with more respiratory-related complications, such as increased hospitalisations in children and adolescents with doctor-diagnosed asthma [36–39], and increased reports of wheezing/whistling symptoms during the winter months [40]. Collectively, these findings underscore the importance of geographical location, as children and adolescents exhibit poorer lung function and more frequent reports of respiratory-related symptoms based on their environmental exposures.

### Mediating Effects of Physical Activity

While we sought to investigate whether physical activity mediates the relationship between air pollution and lung function, the scarcity of studies incorporating physical activity as a variable limited the scope to which this could be assessed [54, 57, 59, 66]. Only four studies integrated a measure of physical activity [54, 57, 59, 66], providing little consensus. For example, Hoek et al. [66] found a small negative association between PEF after training and previous day O_3_ exposure, suggesting that air pollution may have a detrimental effect on lung function when individuals are more vigorously active. Yet, in contrast, Timonen et al. [54] reported no association between various air pollutants and exercise-induced impairment in lung function.

These discrepancies among the findings may be attributable to methodological variations, highlighting the need for more research that is specifically focused on paediatric populations, especially those with asthma. Notably, the use of PEF by Hoek et al. [66] may not have fully captured the complexity of lung function, as decreases in PEF following MVPA or exercise are considered normal, particularly for children with asthma [79]. Indeed, a recent randomised controlled trial by Csonka et al. [80] found that PEF was not a precise predictor of change compared to FEV_1,_ despite the two measures often being used interchangeably as diagnostic tools for asthma.

In the only study to utilise accelerometers, Aguilera et al. [59] reported that during periods of higher air pollution concentrations, children with asthma were less physically active and exhibited more sedentary behaviours. While informative, this study did not establish a clear mediating effect of physical activity on the relationship between air pollution and lung function. Rather, it points more towards behavioural/psychological responses to pollution rather than to physiological mediations. The third study, despite collecting data on the participants’ physical, temporal, and spatial activities (collected via a questionnaire), did not provide results specific to physical activity due to the primary focus being to investigate the effects of air pollution on lung function [57]. This underscores a broader need for more longitudinal studies that focus on assessing the mediating effects of physical activity on air pollution and lung function among children with and without asthma.

Interestingly, two studies that did not meet the inclusion criteria for this review found varied results regarding the interaction between physical activity and lung function, demonstrating the complexity of the potential mediating effect of physical activity on lung function [81, 82]. Specifically, while Turner et al. [82] did not observe any associations between physical activity and increased exposure to ultrafine particulates and lung function, it was highlighted that participants with greater exposure to particulates were 1.3 times more likely to report respiratory symptoms than their counterparts with less exposure. These findings suggest that while this systematic review summarised the negative impact of exposure to PM_2.5_, PM_10_, NO_2_, and O_3_ on children’s lung function, particularly among those with asthma, there is concern regarding ultrafine particulates exacerbating respiratory symptoms. In contrast, Smith et al. [81] failed to find any association between physical activity and lung function, despite the large sample size. Another study attempted to address the impact of air pollution on lung function among children in Poland, reporting PM_10_, SO_2_, and NO_2_ to be of greatest concern. Unfortunately, while this study assessed children’s physical activity via questionnaire, it was not considered within the analysis [83]. A nother study conducted among adults by Laeremans et al. [84] found that, despite exposure to black carbon, long-term physical activity was beneficial to participants' lung function, with FEV_1_ predicted to improve by 0.005 L for every additional weekly hour of physical activity. The findings of Laeremans et al. [84] raise potential questions regarding whether similar benefits might be observed in children.

### Delayed Lag Period

While several of the studies included in this review were of a cross-sectional design (*n* = 4; [63–66]), there were numerous large-scale cohort studies (*n* = 12; [51–62]) which provided a clearer picture of the relationship between air pollution, lung function, and physical activity. Overall, the key findings suggest that there may be longer-term ramifications of air pollution exposure in children with and without asthma [85, 86]. While the duration of this lag period and the associated influencing factors require further research, particularly due to results being of borderline significance, the exposure effect appears to be greatest up to three- or four days post-exposure. Further research is warranted regarding investigating the delayed effect of air pollution on lung function among children.

### Strengths and Limitations

This systematic review comprehensively summarised the available evidence regarding the complex relationship between air pollution, lung function, and physical activity levels in children and adolescents. The review highlights consistent associations, despite not always being statistically significant, regarding a lag effect of air pollution exposure on lung function and provides practical insights for future intervention design. However, the review is hindered by the limited studies available that have considered this relationship, especially regarding physical activity, and considerable inter-study variability in the methods utilised, precluding a meta-analysis from being conducted and necessitating caution when interpreting the synthesised information. Whilst we sought to minimise validity and selection bias by undertaking a quality assessment of each study, these assessments are still subject to the natural bias of the individual(s) reviewing the paper.

### Future directions

This review highlights that longitudinal studies utilising accelerometers are needed to elucidate the potential mediatory or modulatory role of physical activity in the relationship between air pollution and lung function and whether this effect is dependent on age and/or sex. Indeed, there is preliminary evidence to suggest that boys may be more susceptible to the effects of air pollution but whether this is physiologically or behaviourally based (higher physical activity levels), remains to be determined. Comprehensive strategies are needed to further address both environmental and psychological factors when seeking to reduce children’s exposure to, and intake of, air pollution, whilst enhancing and promoting physical activity.

## Conclusions

In conclusion, increased exposure to various air pollutants, particularly O_3,_ PM_2.5,_ and PM_10_, is associated with poorer lung function among children and adolescents living in both urban and rural populations. Children and adolescents with pre-established respiratory conditions, such as asthma, are more vulnerable to the detrimental effects of air pollution exposure. While physical activity may mediate the relationship between air pollution and lung function, there is insufficient evidence to draw conclusions. Given the increasing global dependence on fossil fuels and concurrent escalation of air pollution concentrations, local authorities and governments should continue to seek to reduce air pollution concentrations, and consequently, the burden experienced by people with respiratory conditions, who suffer the consequences of poorer lung function.

## Supplementary Information

Supplementary materials 1.

## Data Availability

Not applicable.

## Abbreviations

FEV_1_: Forced expiratory volume in one second
FeNO: Fractional exhaled nitric oxide
FVC: Forced vital capacity
MMEF: Maximum mid-expiratory flow
MVV: Maximum voluntary ventilation
MVPA: Moderate vigorous physical activity
NO: Nitric oxide
NO_2_: Nitrogen dioxide
NO_x_: Nitrogen oxide
NCD: Noncommunicable disease
O_3_: Ozone
PM_2.5__–__10_: Particulate matter with microns of 2.5 to 10 in diameter
PEF: Peak expiratory flow
WHO: World Health Organization
